# EspF acts as a molecular scaffold to facilitate TRIM25-mediated NLRP3 inflammasome activation during mycobacterial infection

**DOI:** 10.1080/21505594.2026.2723678

**Published:** 2026-09-08

**Authors:** Yang Yang, Yixin Han, Longjian Wu, Xinxin Zang, Wei Xu, Yunjie Li, Ahui Xu, Hexiang Jiang, Jingyan Fan, Luqing Cui, Bei Huang, Guiqing He, Houhui Song

**Affiliations:** a Key Laboratory of Applied Biotechnology on Animal Science & Veterinary Medicine of Zhejiang Province, Zhejiang Engineering Research Center for Veterinary Diagnostics & Advanced Technology, Zhejiang International Science and Technology Cooperation Base for Veterinary Medicine and Health Management, Belt and Road International Joint Laboratory for One Health and Food Safety, China-Australia Joint Laboratory for Animal Health Big Data Analytics, College of Veterinary Medicine of Zhejiang A&F University, Hangzhou, Zhejiang, China; b Department of Infectious Diseases, Laboratory of Infectious Diseases, Wenzhou Central Hospital, Affiliated to Wenzhou Medical University, Wenzhou, China

**Keywords:** *Mycobacterium tuberculosis*, ESX-1 secretion system, EspF, NLRP3 inflammasome, TRIM25, Pyroptosis, host-pathogen interaction

## Abstract

The mycobacterial ESX-1 (Type VII) secretion system is essential for virulence and induces the NLRP3 inflammasome activation. However, the specific bacterial effectors involved in this process, beyond the well-characterized EsxA (ESAT-6), remain largely unidentified. Through systematic screening of ESX-1 effectors, we identified EspF as a potent and evolutionarily conserved activator of the NLRP3 inflammasome across pathogenic mycobacterial species, including *Mycobacterium tuberculosis*, *M. bovis*, and *M. marinum*. Our results demonstrate that EspF significantly augments the mature IL-1β and IL-18 release, ASC speck formation, caspase-1 activation, and gasdermin D (GSDMD)-mediated pyroptosis in THP-1 cells. Notably, infection with *M. smegmatis* or *M. bovis* BCG strain overexpressing EspF significantly enhanced NLRP3 inflammasome activation and pyroptosis, which were completely abolished in NLRP3-deficient cells. Mechanistically, EspF directly interacts with the NACHT and LRR domains of NLRP3. Furthermore, unbiased proteomic screening identified the host E3 ubiquitin ligase TRIM25 as an indispensable binding partner. In THP-1 cells, we confirmed the endogenous interaction and colocalization of the EspF–TRIM25–NLRP3 complex, demonstrating that EspF functions as a molecular scaffold that bridges TRIM25 to NLRP3. This proximity interaction, further validated *in situ* during mycobacterial infection, facilitates TRIM25-mediated K63-linked ubiquitination of NLRP3. *In vivo*, mice infected with *M. bovis* BCG strains overexpressing EspF exhibited exacerbated lung lesions, increased inflammatory cell infiltration, and higher bacterial burdens. Collectively, these findings reveal EspF-TRIM25-NLRP3 axis is a novel mechanism of mycobacterial pathogenesis that drives hyperinflammation to facilitate bacterial survival and dissemination.

## Introduction

Tuberculosis (TB), caused by *Mycobacterium tuberculosis* (Mtb), remains the leading cause of death from a single infectious agent worldwide [1]. In 2024, the World Health Organization reported approximately 10.7 million new TB cases and 1.23 million deaths globally [1]. Despite the deployment of the Bacille Calmette-Guérin (BCG) vaccine and multidrug therapies for decades, the rapid emergence of multidrug-resistant (MDR) and extensively drug-resistant (XDR) Mtb strains continues to pose a major threat to global public health [2,3]. Consequently, a deeper understanding of the host–mycobacterium interaction is crucial to identifying potential targets for host-directed therapies (HDTs) and developing more effective vaccine strategies [4,5].

As a highly adapted intracellular pathogen, Mtb primarily targets alveolar macrophages, where it has evolved elaborate mechanisms to counteract host immune surveillance [6,7]. A critical facet of this host-pathogen interaction involves the activation of the NLRP3 inflammasome, a multi-protein complex that facilitates the maturation of pro-inflammatory cytokines, such as IL-1β and IL-18, and triggers pyroptosis through the cleavage of gasdermin D (GSDMD) [8]. In the context of TB pathogenesis, inflammatory signaling often functions as a “double-edged sword;” while essential for initial bacterial containment, its dysregulation can drive hyperinflammation and extensive tissue damage [9–11]. Therefore, identifying the specific bacterial factors that trigger this pathway is fundamental to the understanding of mycobacterial pathogenesis, as the balance of this inflammatory response ultimately determines whether the infection is resolved or progresses to active disease.

Mycobacterial virulence is heavily dependent on the ESX-1 (Type VII) secretion system, which is essential for phagosomal membrane disruption and the translocation of bacterial effectors into the host cytosol [12–14]. Current evidence identifies that the ESX-1 secretion system is required for triggering the NLRP3 inflammasome activation [15]; however, the molecular understanding remains remarkably limited. While ESAT-6 (6 kDa early secretory antigen target), also called EsxA as per current nomenclature, is the most characterized effector within the ESX-1 system reported to directly activate NLRP3 [16,17], the role of other ESX-1 effectors in NLRP3 activation is poorly understood. Identifying and characterizing these unidentified bacterial modulators is essential to fully elucidate how mycobacteria manipulate host innate immunity to facilitate survival and pathogenesis.

In this study, we identified the conserved ESX-1 effector EspF as a molecular scaffold that recruits the E3 ubiquitin ligase TRIM25 to the NACHT and LRR domains of NLRP3, thereby potentiating inflammasome activation and GSDMD-mediated pyroptosis. Our *in vivo* findings reveal that EspF-driven hyperinflammation exacerbates lung injury and elevates bacterial burden, suggesting that mycobacteria exploit this axis to promote dissemination and survival. These findings provide new insights into the molecular mechanisms of mycobacterial pathogenesis and highlight the EspF-TRIM25-NLRP3 axis as a potential target for host-directed therapies.

## Methods

### Antibodies and reagents

All commercial antibodies were used according to the manufacturers’ instructions. The primary antibodies and their specific working dilutions used in this study were as follows: anti-Flag (ABclonal Technology, AE005, 1:5000), anti-Myc (CST, #2276, 1:1000), anti-HA (CST, #3724, 1:1000), anti-mCherry (Abcam, ab213511, 1:1000), anti-NLRP3 (AdipoGen, AG-20B-0014-C100, 1:1000), anti-caspase-1 (AdipoGen, AG-20B-0048-C100, 1:1000), anti-ASC (Santa Cruz Biotechnology, sc-514414, 1:200), anti-pro-IL-1β (CST, #12703, 1:1000), anti-IL-1β p17 (CST, #83186, 1:1000), anti-TRIM25 (Abcam, ab167154, 1:2000), anti-GFP (ABclonal Technology, AE078, 1:5000), anti-Tubulin (ABclonal Technology, A12289, 1:5000), anti-GAPDH (ABclonal Technology, AC033, 1:100000), and anti-Lamin B1 (HUABIO, SI17-07, 1:2000). The secondary antibodies used for immunoblotting were HRP-conjugated Goat Anti-Rabbit IgG (ABclonal Technology, AS014, 1:5000) and HRP-conjugated Goat Anti-Mouse IgG (ABclonal Technology, AS003, 1:5000). For immunofluorescence confocal microscopy, Goat Anti-Mouse IgG H&L (Alexa Fluor® 488, ab150113), Goat Anti-Rabbit IgG H&L (Alexa Fluor® 555, ab150078), and Donkey Anti-Rabbit IgG H&L (Alexa Fluor® 647, ab150075) secondary antibodies were purchased from Abcam (all used at 1:500). Lipopolysaccharide (LPS, L2630, used at 500 ng/mL), doxycycline (Dox, D9891, used at 100 or 200 ng/mL), and nigericin (N7143, used at 5 μM) were obtained from Sigma-Aldrich.

### Bacterial strains and plasmids

*Mycobacterium smegmatis* was grown in Middlebrook 7H9 (BD Biosciences 271,310) broth supplemented with 0.05% Tween-80 (Sangon Biotech, A500562), or on Middlebrook 7H10 (BD Bioscience 262,710) agar. *M. bovis* BCG (Pasteur 1173P2) was grown in Middlebrook 7H9 broth supplemented with 10% oleic acid-albumin-dextrose-catalase (OADC; Gene Optimal, GOMY0078) and 0.05% Tween-80, or on Middlebrook 7H10 agar supplemented with 10% OADC. *E. coli* DH5α was grown in flasks using lysogeny broth medium at 37°C with antibiotic selection for genetic manipulations. Bacterial expression plasmids were constructed by cloning the fragment of *EspF* (Gene ID: 886,172) into pMN437 (with HA/GFP-tag respectively) vector [18]. The pMN437 vector was a gift from Prof. Michael Niederweis (University of Alabama at Birmingham). For protein expressions in mammalian cells, mycobacterial ESX-1 effectors including *espA* (Gene ID: 885,377), *espB* (Gene ID: 886,214), *espC* (Gene ID: 885,770), *espD* (Gene ID: 885,777), *espE* (Gene ID: 886,185), *espF*, *espJ* (Gene ID: 886,198), *espK* (Gene ID: 886,212), *espR* (Gene ID: 886,184), *PE35* (Gene ID: 886,191), and *PPE68* (Gene ID: 886,201) were cloned into pmCherry-N (with mCherry-tag) vector, human *TRIM25* (NM_005082.5) was cloned into pCMV-Myc-N (with Myc-tag) or pCMV-HA-N (with HA-tag) vector, human *NLRP3* (NM_004895.5), *PYCARD* (ASC, NM_013258.5), *CASP1* (Caspase-1, NM_033292.4), *IL1B* (IL-1β, NM_000576.3) and NLRP3’s mutants were cloned into p3×Flag-CMV7.0 (with Flag-tag) vector. All the plasmids were sequenced at the Sangon Biotech for verification.

### Cell culture and transfection

HEK293T cells were obtained from the China Center for Type Culture Collection (CCTCC) of Wuhan University and cultured in Dulbecco’s modified Eagle’s medium (DMEM; Gibco, C11995500BT) with 10% fetal bovine serum (FBS; Gibco, A5669701). The human monocytic cell line THP-1 cells were also obtained from CCTCC and maintained in RPMI 1640 medium (Gibco, C11875500BT) with 10% FBS. All cells were cultured at 37°C in a humidified 5% CO2 incubator. Transient transfection assays were performed using Lipo8000 (Beyotime Biotechnology, C0533) according to the manufacturer’s instructions.

### Construction of EspF inducible cell line

The lentivirus was produced by co-transfecting HEK293T cells with the inducible expression plasmid pLVXTRE3G-EspF (or the corresponding empty vector as a control), along with the packaging plasmids pMD2G and PSPAX2. The viral supernatant was collected after 48 h incubation, filtered through a 0.45 μm filter. THP-1 cells were incubated with the viral supernatant in the presence of 8 μg/mL polybrene (Solarbio, H8761). After 48 h of infection, cells were selected with 1 μg/mL puromycin (Sigma-Aldrich, P8833) and 0.8 μg/mL G418 (Sigma-Aldrich, A1720) for 72 h. Subsequently, the cells were maintained in a lower concentration of selecting antibiotics (puromycin at 0.5 μg/mL and G418 at 0.4 μg/mL) for continuous culture.

### Generation of knockdown and knock out cell lines

To knock down TRIM25 expression, doxycycline-inducible EspF-expressing THP-1 cells (THP-1-EspF) were transfected with TRIM25-specific or control siRNAs (Genepharma) using Lipofectamine™ RNAiMAX (Invitrogen™, 13,778,075). At 72 h post-transfection, cells were harvested and lysed. The knockdown efficiency was assessed by immunoblotting analysis using an anti-TRIM25 antibody. The sequence of siRNA targeting *TRIM25* is GUCCACCUGAUGUAUAAGUTT. To generate a *TRIM25*-knockout HEK293T cell line, we utilized the CRISPR/Cas9 system. Briefly, sgRNAs specifically targeting the human *TRIM25* gene were designed and cloned into the pSpCas9(BB)-2A-Puro, which co-expresses Cas9 and a puromycin resistance gene. HEK293T cells were seeded in 6-well plates and transfected with the recombinant plasmid using Lipo8000 according to the manufacturer’s protocol. At 24 h post-transfection, cells were subjected to antibiotic selection with 1 μg/mL puromycin for 72 h, until all non-transfected control cells were eliminated. Surviving cells were then sparsely seeded into 96-well plates (0.5 cells/well) for monoclonal colony formation. Gene ablation in clones was verified by sequencing genomic DNA and immunoblotting. The sgRNA sequences for the targeted genes were: GCAAGUUUGACACCAUUUAT.

### Infection of macrophages

THP-1 cells were seeded in 12-well plates at a density of 5 × 10^5^ cells per well and differentiated into macrophages by treatment with 10 ng/mL Phorbol 12-myristate13-acetate (PMA; Sigma-Aldrich, P1585) for 24 h, followed by resting for 24 h. The Mycobacterium strains were grown to mid-log phase (OD_600_ 0.6–0.8) and infected at a multiplicity of infection (MOI) of 10 for 3 h. Following infection, extracellular bacteria were removed by washing the cells three times with warm phosphate-buffered saline (PBS). To kill any remaining extracellular bacteria, cells were then incubated with complete RPMI-1640 medium containing a high dose of 200 µg/mL gentamicin (Sangon Biotech, A506614) for 1 h. After this treatment, cells were washed three additional times with PBS and subsequently maintained in complete medium containing a lower dose of gentamicin (50 µg/mL) for the indicated time periods prior to downstream analyses.

### Cellular fractionation of infected macrophages

THP-1 cells infected with Ms_Vec or Ms_EspF (MOI = 10) were washed three times with 1 × PBS and lysed for 5 min in hypotonic lysis buffer (10 mM HEPES, pH 7.9, 1.5 mM MgCl2, 10 mM KCl, 0.34 M sucrose, 10% glycerol, 0.1% Triton X-100, and 1% protease inhibitor cocktail). Following centrifugation at 1,300 × g for 10 min at 4°C, the supernatant was collected as the macrophage cytosolic fraction. The remaining pellet was resuspended in 1× PBS and sonicated for 5 min to completely lyse the macrophage nuclei and fragment the bacteria. Finally, both the cytosolic fraction and the sonicated pellet were boiled in SDS sample buffer for subsequent immunoblotting analysis.

### Immunoprecipitation and immunoblotting analysis

For immunoprecipitation, HEK293T cells and THP-1 cells were lysed with IP lysis buffer containing 50 mM Tris (pH7.4), 150 mM NaCl, 2 mM EDTA, 10% glycerol and 1% NP-40, supplemented with a protease inhibitor cocktail (Selleck, B14001). Lysates were incubated with appropriate antibody-conjugated magnetic beads at 4°C for 1 h. The beads were then washed five times with cold IP lysis buffer, and the bound proteins were eluted by boiling in SDS loading buffer. For preparation of whole-cell lysates, cells were directly lysed in Radioimmunoprecipitation assay (RIPA) buffer (Beyotime Biotechnology, P0013C) supplemented with a protease inhibitor cocktail, followed by boiling in SDS loading buffer for 10 min. All protein samples were separated by 10%-15% SDS-PAGE gels and transferred onto the PVDF membranes. After blocking with 5% nonfat milk in TBST, the membranes were incubated with designated primary antibodies overnight at 4°C, followed by corresponding HRP-conjugated secondary antibodies. Signals were detected using an enhanced chemiluminescence substrate (NCM Biotech, P10200) and captured by an ECL chemiluminescence imaging analysis system.

### In vitro *pull-down*

Plasmids were individually transfected into HEK293T cells and incubated for 24 h. The cells were lysed with IP lysis buffer supplemented with a protease inhibitor cocktail. The supernatants were incubated with beads coated with specific antibodies at 4°C for 2 h. Flag-tagged proteins were competitively eluted from the Flag beads using Flag peptide (Sigma, SAE0194) at a concentration of 100 μg/mL for 2 h at 4°C. The eluates were subsequently incubated with beads conjugated with either mCherry- or Myc-tagged proteins at 4°C overnight. Finally, the beads were washed and subjected to immunoblotting analysis.

### Immunofluorescence confocal microscopy

THP-1 or HEK293T cells were seeded on coverslips placed in 24 well plates at a density of 5 × 10^5^ cells per well. Following the relevant experiments, cells were fixed with 4% paraformaldehyde at room temperature for 30 min. After fixation, cells were permeabilized and blocked by incubation with blocking buffer for 1 h at room temperature. Cells were then incubated overnight at 4°C with appropriate primary antibodies, followed by incubation with the corresponding fluorescent-conjugated secondary antibodies for 1 h at room temperature. Nuclei were counterstained with 4,6-diamidino-2-phenylindole (DAPI, C1002, Beyotime) for 10 min. The stained cells were observed using a laser scanning microscope (SpinSR, Olympus). For the quantification of ASC specks, randomly selected fields for each experimental condition were captured. The total number of cells in each field was determined by counting DAPI-stained nuclei, and the number of cells containing a distinctly condensed ASC speck was counted manually using ImageJ software. Approximately 100 cells were analyzed per condition in each independent experiment. For colocalization analysis, the Pearson correlation coefficient was calculated using ImageJ software.

### Proximity ligation in situ assay

The proximity ligation assay (PLA) was performed using Duolink In Situ Detection Reagents Red (Sigma-Aldrich, DUO92008) according to the manufacturer’s protocol. Briefly, THP-1 cells (1 × 10^5^) grown on poly-L-lysine coverslips were infected with Ms_EspF or Ms_Vec at a MOI of 10 for 48 h. After standard fixation and permeabilization, cells were blocked with Duolink Blocking Solution for 1 h at 37°C, and then incubated with mouse anti-NLRP3 and rabbit anti-TRIM25 antibodies for 1 h at 37°C. After two washes, cells were incubated with PLA probes (Anti-Mouse PLUS and Anti-Rabbit MINUS, Sigma-Aldrich, DUO92002 and DUO92004) for 1 h at 37°C to allow specific hybridization of adjacent targets (<40 nm). Subsequent ligation (30 min) and amplification (90 min, in the dark) steps were performed at 37°C. Finally, coverslips were mounted with DAPI and analyzed by confocal microscopy.

### Lactate dehydrogenase (LDH) assay

The supernatants of cells were collected and analyzed for LDH activity using the CytoTox 96 Non-Radioactive Cytotoxicity Assay kit (Promega, G1780), following the manufacturer’s manual.

### ELISA

Supernatants were harvested at the indicated time points following stimulation. Concentrations of IL-1β and IL-18 were determined using commercial ELISA kits (IL-1β: R&D Systems, DY201; IL-18: ABclonal Technology, RK00176) according to the manufacturers’ protocols.

### In vivo *infection*

Specific-pathogen-free (SPF) female C57BL/6 mice aged 6–8 weeks were purchased from Vital River Laboratory Animal Technology Co., Ltd. Animals were housed in a controlled environment and allowed to acclimatize for 7 days prior to the initiation of experiments. Based on established protocols for assessing lung bacterial burden and pathology [19], a sample size of *n* = 5 per group was selected to ensure sufficient statistical power. Mice were randomly assigned to three experimental groups using a computer-generated random number sequence: (1) Control group; (2) BCG_Vec infected group; (3) BCG_EspF infected group. No inclusion or exclusion criteria were set after the start of the study, and no animals were excluded from the final analysis. The mice were subjected to aerosol-infection with PBS, BCG_Vec or BCG_EspF at 200 CFUs, respectively. All mice were euthanized with isoflurane inhalation in their home cages in accordance with the AVMA guidelines at 30 days post infection. The left lung was successively isolated, photographed, and homogenized with PBST. Serial dilutions were spread onto 7H10 plates and incubated for 3–4 weeks to determine the bacterial load, which was used as the key indicator for evaluating mycobacterial survival. The right lung was fixed with 10% formalin for hematoxylin and eosin (H&E) staining and acid-fast staining, respectively. The study was approved by the Animal Experimentation Ethics Committee of Zhejiang A&F University (No. AFUAC202586) and in accordance with ARRIVE guidelines.

### Statistical analysis

All assays were performed at least three times independently. Data are presented as mean ± SD and analyzed using GraphPad Prism 8. Statistical significance was determined by an unpaired two-tailed Student’s *t*-test for two-group comparisons, or by one-way or two-way ANOVA followed by Tukey’s or Sidak’s multiple comparisons test for multi-group analyses. Statistical significance was defined as * *p* < 0.05, *p* < 0.01, and *** *p* < 0.001; *ns*, not significant.

## Results

### EspF promotes NLRP3 inflammasome activation and pyroptosis

**Figure 1.**
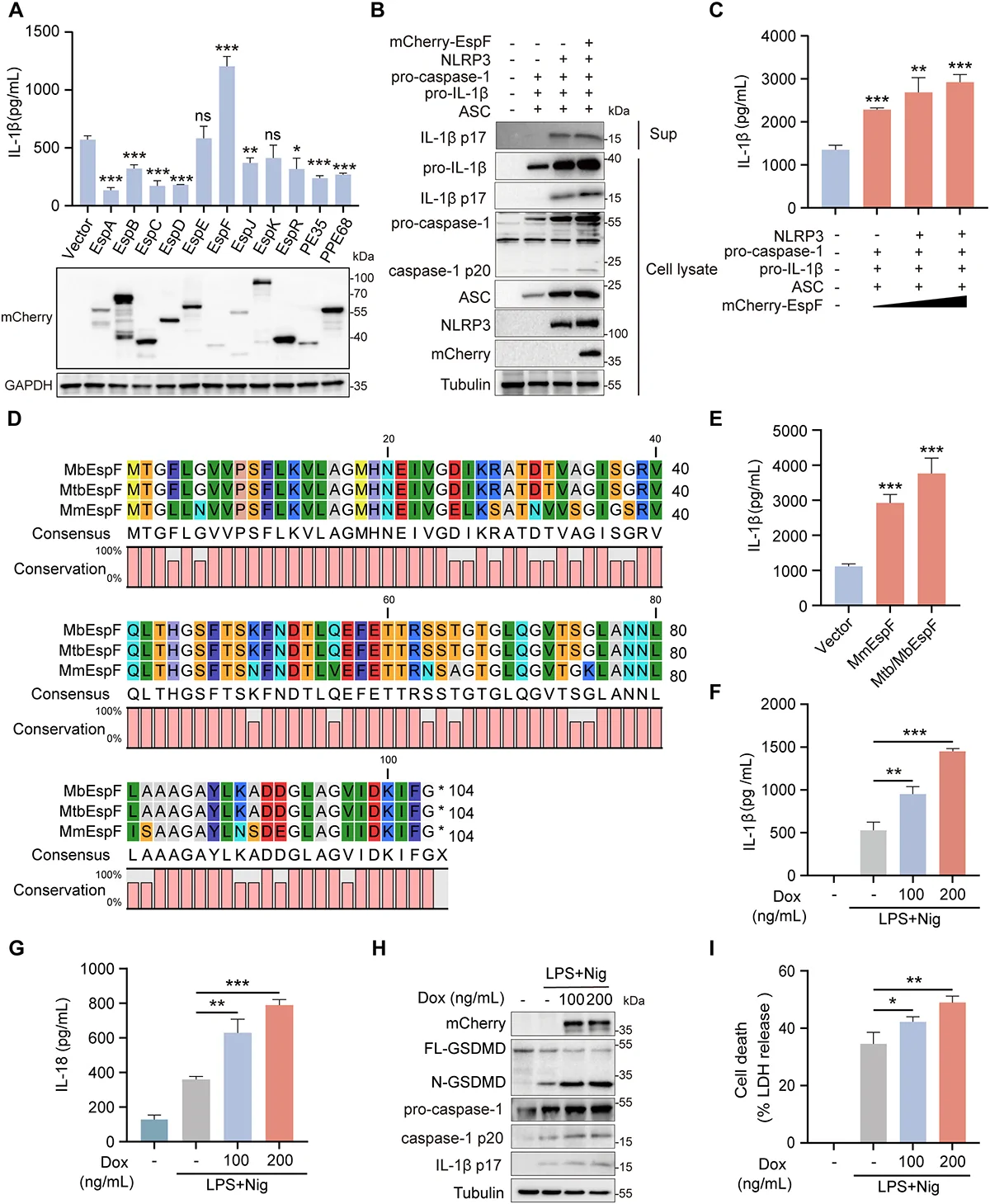
EspF triggers NLRP3 inflammasome activation and pyroptosis. (A) HEK293T cells were co-transfected with plasmids encoding NLRP3, ASC, pro-caspase-1, or pro-IL-1β and with plasmids encoding mCherry-tagged EspA, EspB, EspC, EspD, EspE, EspF, EspJ, EspK, EspR, PE35, or PPE68 for 24 h. (Top) Supernatants were analyzed by ELISA for IL-1β. (Bottom) Cell lysates were analyzed by immunoblotting. (B) HEK293T cells were co-transfected with plasmids encoding NLRP3, ASC, pro-caspase-1, and pro-IL-1β and with plasmid encoding mCherry-tagged EspF for 24 h. Cell lysates were analyzed for cleavage of pro-caspase-1 and GSDMD by immunoblotting. (C) HEK293T cells were co-transfected with plasmids encoding NLRP3, ASC, pro-caspase-1, and pro-IL-1β and with different concentrations of plasmid encoding mCherry-tagged EspF for 24 h. Supernatants were analyzed by ELISA for IL-1β. (D) Alignment of protein sequences of EspF from Mtb (MtbEspf), *M. bovis* (MbEspf), and *M. marinum* (MmEspf). (E) HEK293T cells were co-transfected with plasmids encoding NLRP3, ASC, pro-caspase-1, and pro-IL-1β and with plasmid encoding mCherry-tagged Mtb/MbEspF or MmEspF for 24 h. Supernatants were analyzed by ELISA for IL-1β. (F-I) the DOX-inducible EspF expressing THP-1 cells were pretreated with or without indicated concentrations of doxycycline for 72 h, then primed with 500 ng/mL LPS for 4 h and stimulated with 5 μM nigericin for 1 h. The secretion of IL-1β (F) and IL-18 (G) in the supernatant, protein levels of caspase-1 and GSDMD (H), and cytotoxicity (I) were measured. Data are presented as mean ± SD of three independent experiments. Statistical significance was determined by one-way ANOVA followed by Tukey’s multiple-comparison test (A, C, E, F, G, and I). *p < 0.05, **p < 0.01, and ***p < 0.001. ns, not significant.

To identify potential bacterial effectors within the ESX-1 secretion system that activate the NLRP3 inflammasome, we co-transfected HEK293T cells with 11 known ESX-1 effectors together with the core NLRP3 inflammasome components (NLRP3, ASC, pro-caspase-1, and pro-IL-1β). The secretion of mature IL-1β into the culture supernatant was then measured to assess inflammasome activation. Notably, EspF exhibited the strongest activating effect, eliciting the highest level of IL-1β release among all the effectors tested (Figure 1(A)). Subsequent immunoblotting confirmed that EspF significantly enhanced IL-1β maturation and caspase-1 activation (Figure 1(B)). Moreover, EspF was found to promote NLRP3 inflammasome activation in a dose-dependent manner (Figure 1(C)).

Given that *Mycobacterium tuberculosis* (Mtb), *Mycobacterium bovis* (*M. bovis*), and *Mycobacterium marinum* (*M. marinum*) are known to induce pyroptosis via the ESX-1 secretion system, we analyzed the evolutionary conservation of EspF using CLC Sequence Viewer. Sequence alignment revealed that EspF from *M. bovis* (MbEspF) is identical to its homolog in Mtb (MtbEspF), while sharing 78.6% sequence identity with the version found in *M. marinum* (MmEspF) (Figure 1(D)). Notably, MmEspF also demonstrated the ability to promote NLRP3 inflammasome activation in our HEK293T reconstitution model (Figure 1(E)), suggesting that EspF-mediated inflammasome activation is a conserved mechanism across pathogenic mycobacterial species.

To corroborate these findings in a physiologically relevant immune cell model, we generated a doxycycline (Dox)-inducible EspF expression system in THP-1 cells. We evaluated IL-1β release in these THP-1-EspF macrophages in the presence or absence of Dox induction under different stimulation conditions. Notably, inducing EspF expression alone in the presence of PBS or LPS did not significantly promote IL-1β production. EspF significantly enhanced IL-1β release only when cells were co-stimulated with LPS and nigericin (Figure S1A and Figure S1F). Under these co-stimulation conditions, EspF amplified NLRP3 inflammasome activation, as evidenced by increased release of IL-18, enhanced caspase-1 activation, augmented ASC speck formation, and increased cleavage of gasdermin D (GSDMD), culminating in elevated levels of pyroptosis (Figure 1(G–I) and Figure S1B-C). Collectively, these data indicate that EspF functions as a potent bacterial effector and enhancer that amplifies canonical NLRP3 signaling and subsequent pyroptosis once initiated.

### EspF augments NLRP3 inflammasome activation during mycobacterial infection

**Figure 2.**
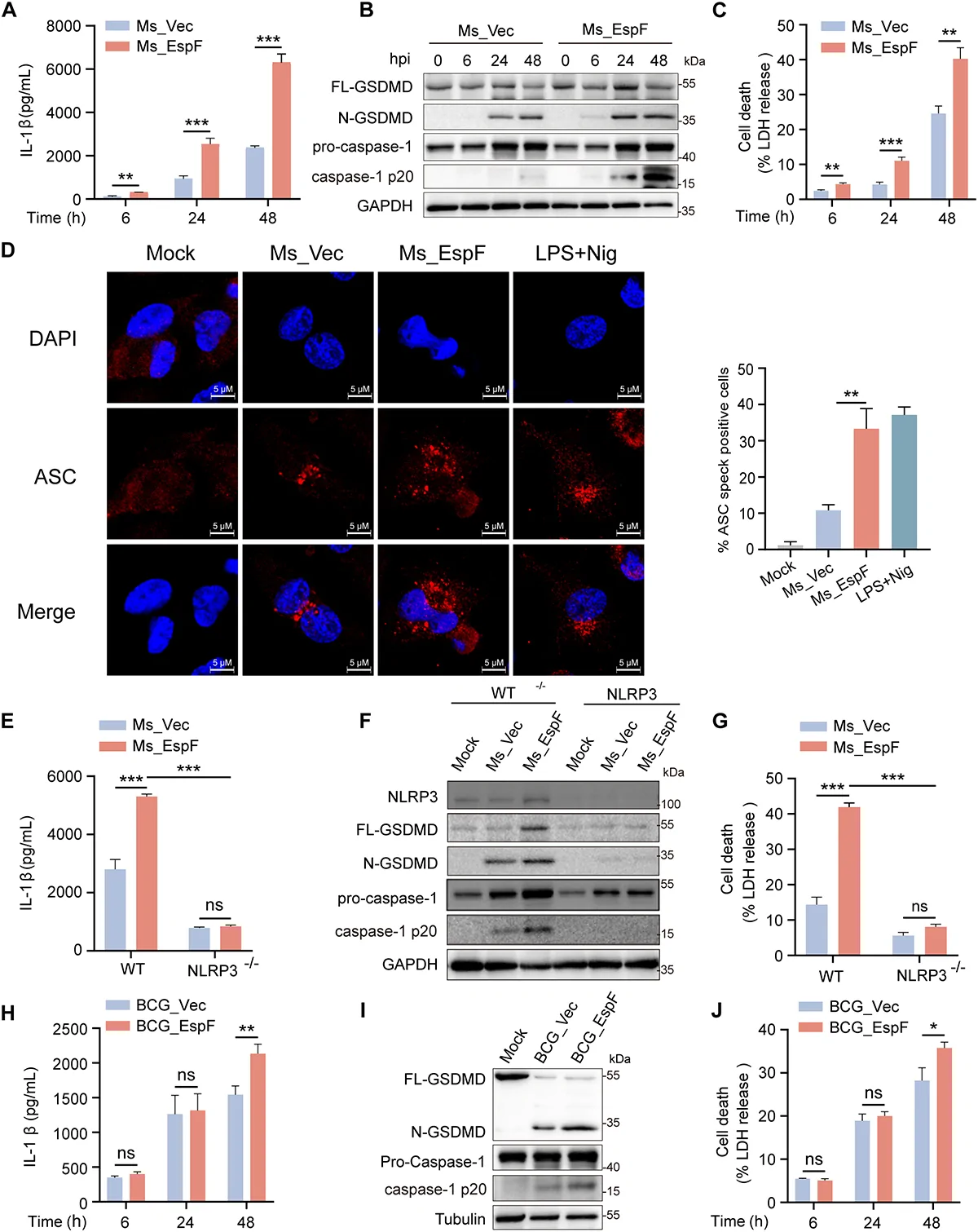
EspF triggers NLRP3 inflammasome activation and pyroptosis during mycobacterial infection. (A-D) THP-1 cells were infected with recombinant *M. smegmatis* expressing EspF (Ms_espf) or the control (Ms_vec) at MOI of 10 for the indicated time. (A) IL-1β secretion in the supernatant was measured by ELISA. (B) Protein levels of pro-caspase-1, cleaved caspase-1, GSDMD, and the GSDMD N-terminal fragment (N-GSDMD) in cell lysates were detected by immunoblotting. (C) cytotoxicity was determined by LDH release assay. (D) Formation of ASC specks was assessed by immunofluorescence at 48 h post-infection. As a positive control, THP-1 cells were primed with 500 ng/mL LPS for 4 h followed by stimulation with 5 μM nigericin for 1 h. Scale bars, 5 μm. The percentage of cells containing ASC specks was quantified from randomly selected fields using ImageJ software. Approximately 100 cells were examined. (E-G) the secretion of IL-1β in the supernatant (E), protein levels of caspase-1 and GSDMD (F), and cytotoxicity (G) were measured in WT and *NLRP3*-deficient THP-1 cells infected with Ms_EspF or (Ms_vec) at an MOI of 10 for 48 h. (H-J) the secretion of IL-1β in the supernatant (H), protein levels of caspase-1 and GSDMD (I), and cytotoxicity (J) were measured in THP-1 cells infected with recombinant BCG at an MOI of 10 for the indicated time. Data are presented as mean ± SD of three independent experiments. Statistical significance was determined by one-way ANOVA followed by Tukey’s multiple comparisons test (D) or two-way ANOVA followed by Tukey’s multiple comparisons test (A, C, E, G, H, and J). *p < 0.05, **p < 0.01, and ***p < 0.001. ns, not significant.

To evaluate the relevance of EspF during bacterial pathogenesis, we generated an EspF-overexpressing *M. smegmatis* strain (Ms_EspF). Ms_EspF exhibited growth kinetics and colony morphology comparable to the empty vector control (Ms_Vec; Figure S2A-C). Importantly, subcellular fractionation confirmed that EspF is effectively translocated into the host cell cytosol during infection (Figure S2G). In comparison with Ms_Vec infection, Ms_EspF induced significantly higher levels of IL-1β secretion, caspase-1 activation, GSDMD cleavage, and cell death (Figure 2(A–C)). Notably, ASC oligomerization, a critical step in inflammasome assembly, was significantly increased in Ms_EspF-infected cells (Figure 2(D)). Crucially, all Ms_EspF-induced effects were completely abolished in NLRP3-deficient THP-1 cells, confirming that EspF-mediated response is strictly NLRP3-dependent (Figure 2(E–G)). We further examined the role of EspF in a more physiologically relevant *M. bovis* BCG model (Figure S2D-F). At 48 h post-infection, BCG_EspF induced higher levels of IL-1β secretion, caspase-1 activation, and pyroptosis compared to the BCG_Vec control (Figure 2(H–J)). These results establish EspF as a bacterial effector that augments NLRP3 inflammasome activation during mycobacterial infection.

### EspF interacts directly with the NACHT and LRR domains of NLRP3

**Figure 3.**
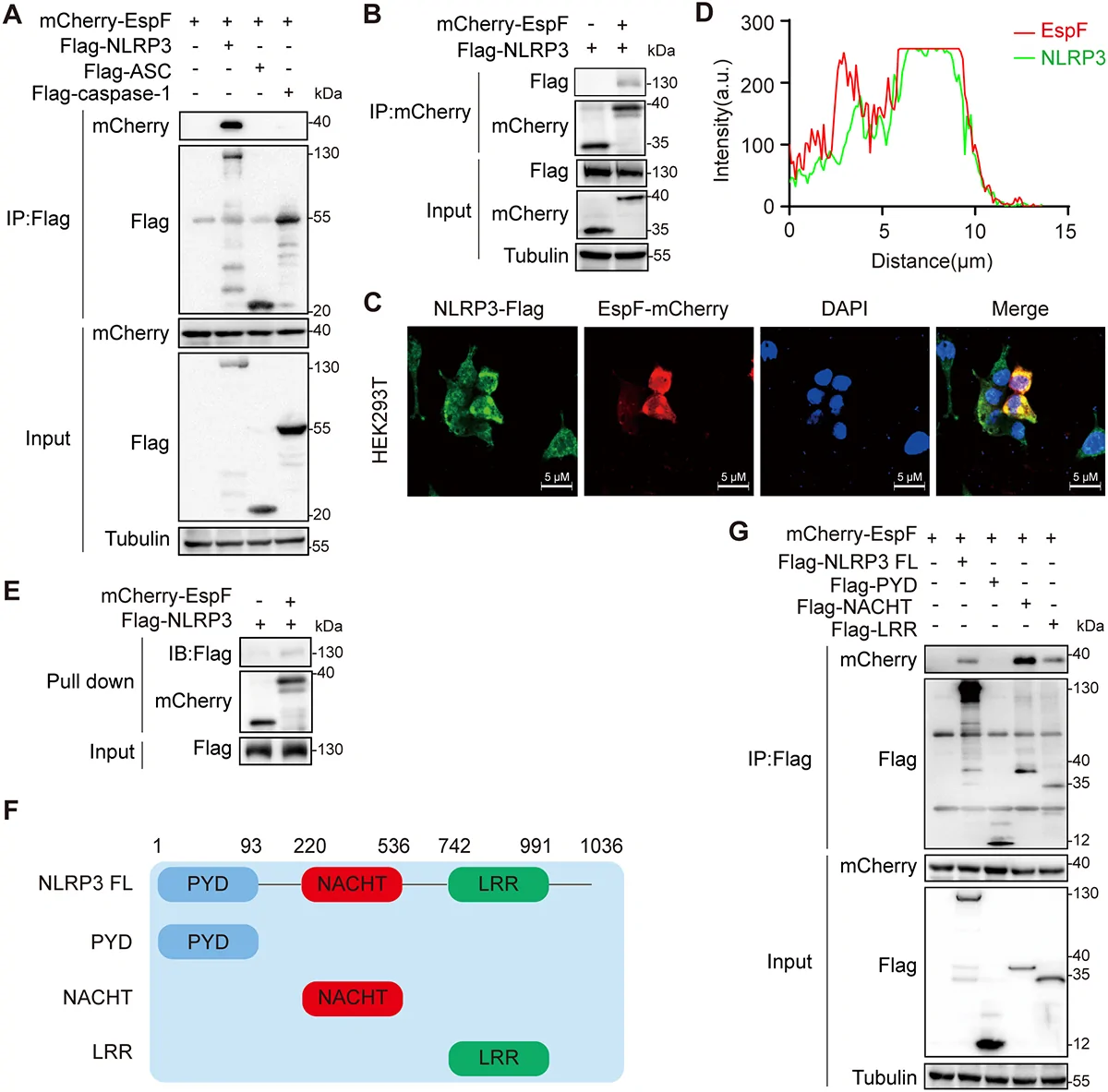
EspF interacts with NLRP3. (A) HEK293T cells were co-transfected with mCherry-EspF and Flag-NLRP3, ASC, or pro-caspase-1 for 24 h. Cell lysates were immunoprecipitated with anti-Flag-M2 beads and detected with indicated antibodies by immunoblotting. (B) lysates from HEK293T cells expressing mCherry-EspF and Flag- NLRP3 were immunoprecipitated with an anti-mCherry antibody. (C-D) HEK293T cells transfected with mCherry-EspF and Flag-NLRP3 were fixed and stained with an anti-Flag antibody and an appropriate secondary antibody. (C) Representative confocal microscopy images showing the intracellular colocalization of EspF (red) and NLRP3 (green). Scale bars, 5 μm. (D) Line-scan profile across the cell in (C) depicting the spatial overlap of fluorescence intensity for EspF and NLRP3. (E) HEK293T cells were transfected with mCherry-EspF, mCherry-tagged Empty Vector, and Flag-NLRP3 for 24 h, respectively. All proteins were enriched using beads with specific antibodies. The Flag-NLRP3 were eluted from Flag beads with Flag peptide and subsequently incubated with immobilized mCherry-tagged proteins on mCherry-beads. The coimmunoprecipitated Flag proteins were detected using an anti-Flag antibody. (F) Schematic diagram of NLRP3 and its truncated mutants. (G) HEK293T cells were co-transfected with mCherry-EspF and Flag-NLRP3 or its mutants. Lysates were immunoprecipitated with an anti-Flag antibody and analyzed by immunoblotting. All immunoblots and micrographs are representative of three independent experiments.

To elucidate the molecular mechanism by which EspF activates the inflammasome, we examined potential interactions between EspF and core NLRP3 inflammasome components. We co-transfected HEK293T cells with mCherry-tagged EspF and Flag-tagged NLRP3 inflammasome components, followed by co-immunoprecipitation (Co-IP). EspF strongly interacted with NLRP3 but showed no detectable binding to ASC or pro-caspase-1 (Figure 3(A)). This interaction was further confirmed by reciprocal immunoprecipitation and confocal microscopy, which demonstrated robust colocalization between EspF and NLRP3 in the cytoplasm (Figure 3(B–D)). To determine if the interaction between EspF and NLRP3 was direct, we performed an *in vitro* pull-down assay using purified recombinant proteins from HEK293T cells, which confirmed a direct physical association between EspF and NLRP3 (Figure 3(E)). Mapping of the interaction domains revealed that EspF binds specifically to the NACHT and LRR domains of NLRP3, but not the N-terminal PYD domain (Figure 3(F,G)). These data collectively indicate that EspF directly binds to NLRP3 by interacting with the NACHT and LRR domains.

### TRIM25 is indispensable for EspF-induced NLRP3 inflammasome activation

**Figure 4.**
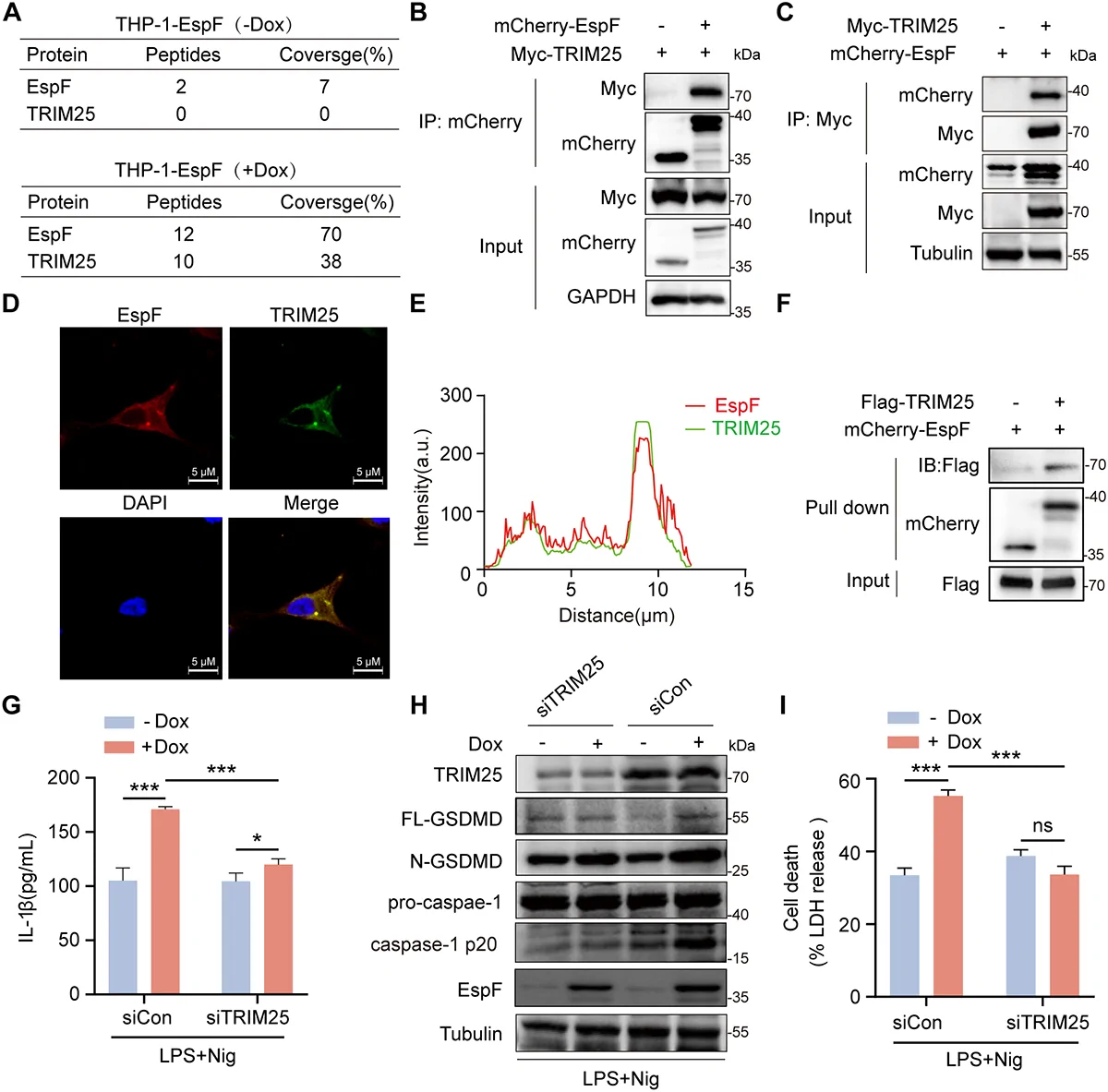
NLRP3 inflammasome activation by EspF is strictly dependent on TRIM25. (A) Mass spectrometry analysis identified TRIM25 as a potential interactor with EspF. (B) HEK293T cells were co-transfected with mCherry-EspF and Myc-TRIM25 for 24 h. Cell lysates were immunoprecipitated with an anti-mCherry antibody and detected with the indicated antibodies by immunoblotting. (C) lysates from HEK293T cells expressing mCherry-EspF and Myc-TRIM25 were immunoprecipitated with an anti-Myc antibody. (D) Colocalization between exogenous EspF and TRIM25 was examined in HEK293T cells by Confocal microscopy. (E) Line scan graphs depicting the co-localization of EspF (red) with TRIM25 (green) in (D). (F) An *in vitro* pull-down assay to confirm the direct interaction between TRIM25 and EspF. (G-I) the Dox-inducible EspF expressing THP-1 cells were pretreated with or without doxycycline and transfected with siRNA targeting *TRIM25* (si-TRIM25) or negative control siRNA (si-NC) for 72 h, then primed with 500 ng/mL LPS for 4 h and stimulated with 5 μM nigericin for 1 h. The secretion of IL-1β (G) in the supernatant, protein levels of caspase-1 and GSDMD (H), and cytotoxicity (I) were measured. Data are presented as mean ± SD of three independent experiments. Statistical significance was determined by two-way ANOVA followed by Tukey’s multiple comparisons test (G and I). All immunoblots and micrographs are representative of three independent experiments. *p < 0.05, ***p < 0.001, ns, not significant.

To identify cellular partners of EspF that involved in the interaction with the inflammasome, we performed an unbiased proteomic screen using liquid chromatography-tandem mass spectrometry (LC-MS/MS) on EspF immunoprecipitants from THP-1 cells (Figure 4(A)). The E3 ubiquitin ligase TRIM25 was identified as a high-confidence candidate interactor. The EspF-TRIM25 association was validated via co-immunoprecipitation in HEK293T cells and corroborated by confocal fluorescence microscopy, which showed clear cytoplasmic colocalization (Figure 4(B–E)). Moreover, an *in vitro* pull-down assay also confirmed the direct interaction between EspF and TRIM25 (Figure 4(F)). To determine the role of TRIM25 in EspF-induced NLRP3 inflammasome activation, we silenced TRIM25 expression with TRIM25-specific small-interfering RNA (siRNA) and assessed NLRP3 inflammasome activation after LPS primed nigericin stimulation in Dox-inducible EspF expressing THP-1 cells. TRIM25 knockdown remarkably attenuated IL-1β secretion, caspase-1 activation, GSDMD cleavage, and cell death (Figure 4(G–I)). Collectively, these data demonstrate that TRIM25 is required for EspF-induced NLRP3 inflammasome activation in macrophages.

### EspF recruits TRIM25 to promote K63-linked NLRP3 ubiquitination

**Figure 5.**
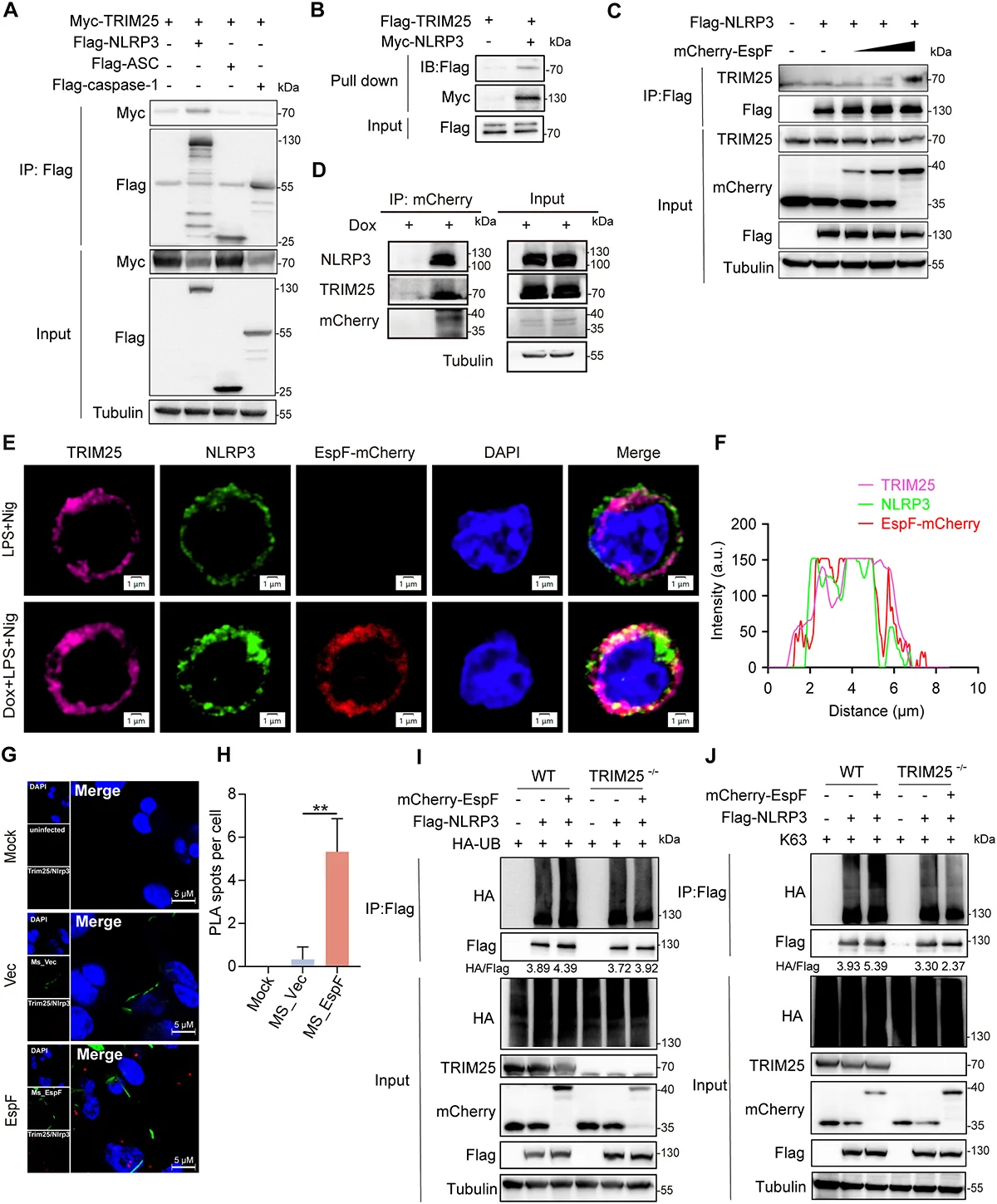
EspF enhances the association between TRIM25 and NLRP3. (A) HEK293T cells were co-transfected with Myc-TRIM25 together with Flag-NLRP3, Flag-ASC, or Flag-caspase-1 for 24 h. Cell lysates were immunoprecipitated with anti-Flag M2 beads and detected with the indicated antibodies by immunoblotting. (B) An *in vitro* pull-down assay using Flag-TRIM25 and Myc-NLRP3 confirming the direct physical interaction between TRIM25 and NLRP3. (C) HEK293T cells were co-transfected with Flag-NLRP3, TRIM25, and an increasing dose of mCherry-EspF. Cell lysates were immunoprecipitated with anti-Flag M2 beads and detected with the indicated antibodies by immunoblotting. (D) Dox-inducible EspF-expressing THP-1 cells were pretreated with doxycycline for 72 h to induce EspF expression, then primed with 500 ng/mL LPS for 4 h and stimulated with 5 μM nigericin for 1 h. Cell lysates were immunoprecipitated with an anti-mCherry antibody or control IgG and detected with the indicated antibodies by immunoblotting. (E) Intracellular colocalization of TRIM25 (magenta), NLRP3 (green), and EspF (red) was examined by confocal microscopy. (F) Line-scan profile across the cell in (E) depicting the spatial overlap of fluorescence intensity for TRIM25, NLRP3, and EspF. (G) THP-1 cells were infected with GFP-expressing recombinant Ms_EspF or Ms_Vec at an MOI of 10 for 24 h, and subjected to proximity ligation assay (PLA) to detect endogenous TRIM25-NLRP3 interactions. Green fluorescence indicates the presence of GFP-tagged *M. smegmatis* (Ms_vec or Ms_EspF). Each red spot represents a distinct PLA signal indicating proximity between TRIM25 and NLRP3 (<40 nm). Nuclear staining with DAPI (blue) was used to determine the average number of PLA spots per cell. Scale bars, 5 µm. (H) quantification of PLA spots per cell in THP-1 cells treated as in (G). Approximately 100 cells were examined per condition across three independent experiments. (I) Wild-type (WT) and TRIM25-deficient HEK293T cells were co-transfected with mCherry-EspF, Flag-NLRP3, and HA-tagged ubiquitin (HA-Ub). Cell lysates were immunoprecipitated with anti-Flag M2 beads and detected with the indicated antibodies by immunoblotting. (J) WT and TRIM25^−/−^ HEK293T cells were co-transfected with mCherry-EspF, Flag-NLRP3, and HA-tagged K63-only ubiquitin (HA-K63 Ub). Cell lysates were immunoprecipitated with anti-Flag M2 beads and analyzed by immunoblotting. Data in (H) are presented as mean ± SD of three independent experiments. Statistical significance was determined by an unpaired two-tailed Student’s *t*-test (H). All immunoblots and micrographs are representative of three independent biological replicates. **p < 0.01.

Given that both NLRP3 and TRIM25 interact with EspF, we investigated whether TRIM25 itself is a novel NLRP3-binding partner. Co-IP assays revealed that TRIM25 strongly interacts with NLRP3, but not with ASC or pro-caspase-1 (Figure 5(A) and Figure S3A), a finding supported by their intracellular colocalization (Figure S3B,C). An *in vitro* pull-down assay also confirmed the direct interaction between TRIM25 and NLRP3 (Figure 5(B)). We further proved that the full-length NLRP3, domains of NACHT and LRR, but not PYD domain, interact with TRIM25 (Figure S3D). We then hypothesized that EspF might function as a molecular scaffold to promote this interaction. Notably, the interaction between TRIM25 and NLRP3 increased in a dose-dependent manner with increasing expression of EspF in HEK293T cells (Figure 5(C)). To determine if these interactions naturally occur during active inflammasome signaling, we utilized THP-1 macrophages expressing DOX-inducible EspF. Following DOX induction and subsequent stimulation with LPS and nigericin, Co-IP assays demonstrated that EspF interacts with endogenous TRIM25 and NLRP3 (Figure 5(D)). Furthermore, immunofluorescence staining confirmed the distinct spatial colocalization of EspF, TRIM25, and NLRP3 within these macrophages (Figure 5(E,F)). While immunofluorescence indicates spatial overlap, we further employed a proximity ligation assay (PLA) to directly assess the precise physical interaction between TRIM25 and NLRP3 in the context of actual mycobacterial infection. Consistent with our scaffold model, cells infected with Ms_EspF exhibited a significantly higher number of TRIM25-NLRP3 interaction foci compared to those infected with Ms_Vec (Figure 5(G,H)). Collectively, these findings provide direct evidence that EspF functions as a molecular bridge for the TRIM25-NLRP3 complex during infection.

Given that TRIM25 is an E3 ubiquitin ligase, we next investigated whether EspF recruits TRIM25 to facilitate NLRP3 ubiquitination. As shown in Figure 5(I), EspF promotes NLRP3 ubiquitination, which was abrogated when TRIM25 was knocked out by CRISPR-Cas9. It is reported that TRIM25 could promote K63-linked substrate ubiquitination [20]. Indeed, EspF-mediated K63-linked NLRP3 ubiquitination was significantly decreased in the absence of TRIM25 (Figure 5(J)). These findings suggest that EspF facilitates TRIM25-mediated K63-linked NLRP3 ubiquitination, thereby augmenting inflammasome signaling.

### *EspF promotes mycobacteria survival* in vivo

**Figure 6.**
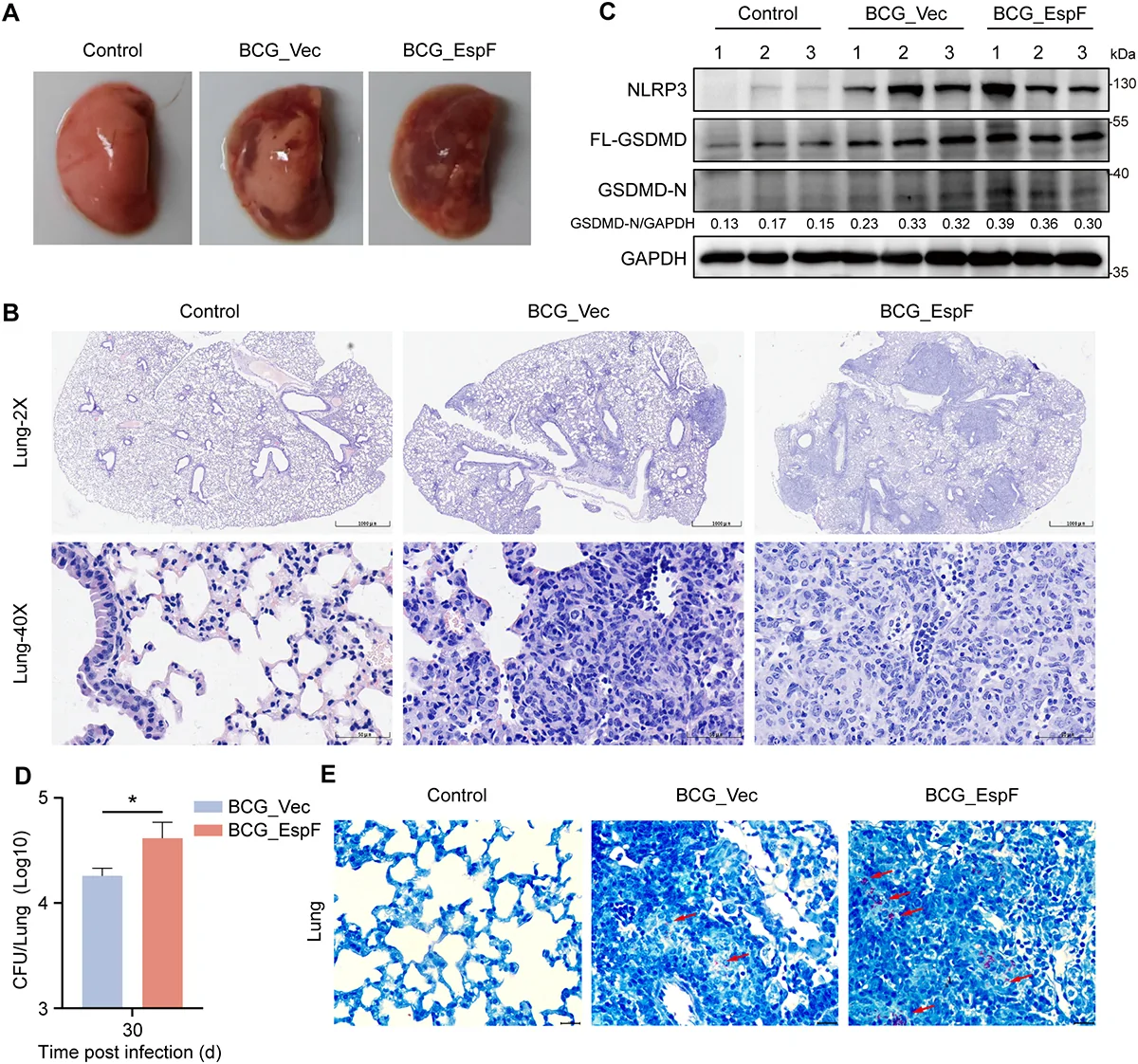
EspF promotes mycobacteria survival *in vivo*. C57BL/6 mice were infected with recombinant BCG strains BCG_EspF or BCG_Vec at ~200 CFUs via the intranasal route. At 30 days post-infection, animals were euthanized, and lungs were collected. (A) Representative images of the lung showed the gross pathological changes of uninfected and recombinant BCG strains infected mice. (B) Representative hematoxylin and eosin (H&E)-stained lung sections. (C) immunoblotting analysis of NLRP3 expression, GSDMD cleavage in the lung tissue lysates (*n* = 3 individual mice per group). (D) Mycobacterial load in the lungs was determined by plating lung homogenates on 7H10 agar plates. (E) Representative acid-fast stained lung sections (red arrows indicate acid-fast bacilli). Data are presented as mean ± SD (n=5 mice per group). Statistical significance was determined by an unpaired two-tailed Student’s*t*-test (D). All gross pathology images and histological sections are representative of at least three independent experiments. *p < 0.05; ns, not significant.

To explore the role of the EspF protein in the pathogenicity of mycobacterium, C57BL/6 mice were aerosol-infected with roughly 200 colony-forming units (CFUs) of recombinant BCG strains BCG_EspF or BCG_Vec for 30 days. Gross pathological observations revealed that EspF expression exacerbated the development of lesions in the lungs of infected mice (Figure 6(A)). Histopathological analysis of the inflammatory area in the right lung lobe of infected mice showed that EspF significantly increased lung inflammation (Figure 6(B)). Moreover, the lung tissue from mice infected with BCG_EspF showed more GSDMD cleavage (Figure 6(C)). Additionally, the bacterial burden in the lung infected with BCG_EspF was higher than that in the lung infected with BCG_Vec (Figure 6(D)). Qualitative analysis using acid-fast staining in the lung yielded similar results at 30 days after challenge (Figure 6(E)). These results demonstrate that EspF could promote mycobacteria dissemination and colonization and induce organ pathological injury *in vivo*.

## Discussion

**Figure 7.**
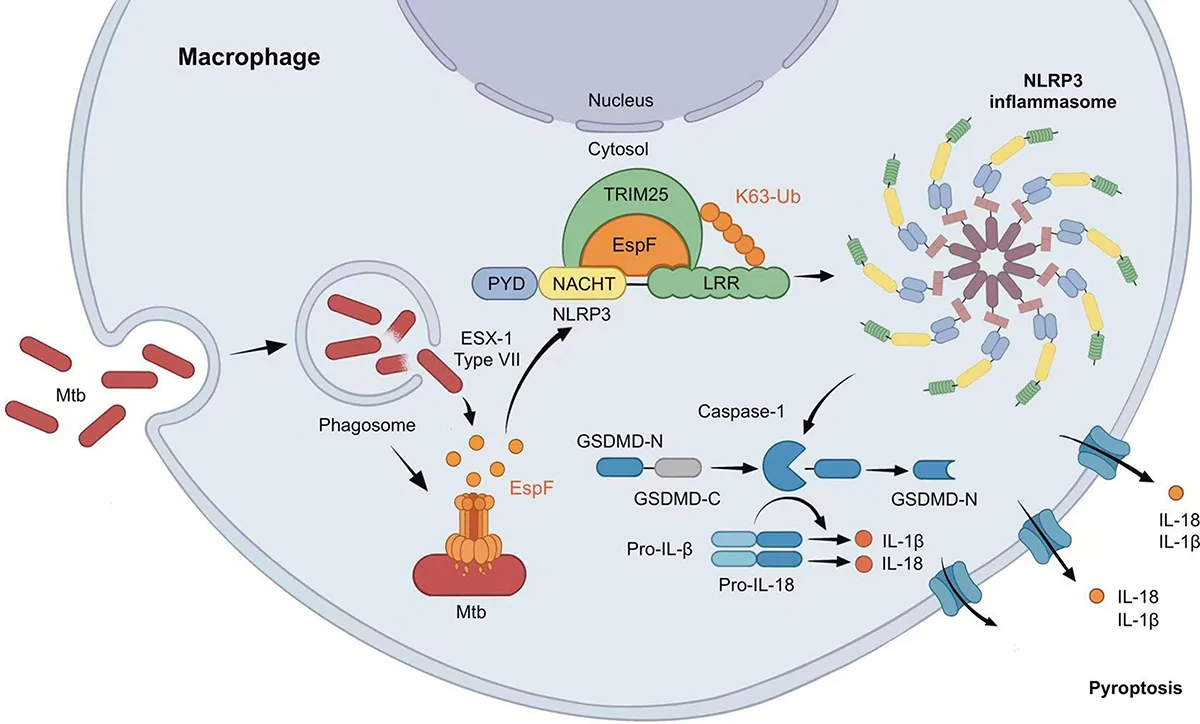
A proposed model for the EspF-TRIM25-NLRP3 axis in mycobacterial pathogenesis. During mycobacterial infection,*M. tuberculosis* (Mtb) resides within the phagosome of the macrophage. The ESX-1 (Type VII) secretion system facilitates the translocation of the bacterial effector EspF into the host cytosol. Once in the cytosol, EspF acts as a molecular scaffold by directly binding to the NACHT and LRR domains of NLRP3. This interaction facilitates the recruitment of the host E3 ubiquitin ligase TRIM25 to the NLRP3 complex. TRIM25 subsequently mediates the K63-linked ubiquitination of NLRP3, which potentiates the assembly and activation of the NLRP3 inflammasome. Activation of the inflammasome leads to the recruitment and activation of caspase-1, which cleaves gasdermin D (GSDMD) into its active N-terminal fragment (GSDMD-N) and processes pro-IL-1β and pro-IL-18 into their mature forms. The GSDMD-N fragments form pores in the plasma membrane, resulting in pyroptosis and the release of mature IL-1β and IL-18.

Tuberculosis remains one of the most challenging infectious diseases worldwide, largely due to the extraordinary capacity of Mtb to manipulate host immune responses to long-term persistence [7,21]. In this study, we identify EspF as a previously unrecognized ESX-1–secreted effector that functions as a conserved activator of the NLRP3 inflammasome. Our findings reveal a mechanism by which EspF directly engages NLRP3 and orchestrates the recruitment of the E3 ubiquitin ligase TRIM25, thereby amplifying inflammasome activation, GSDMD–mediated pyroptosis, and inflammatory pathology that ultimately favors mycobacterial survival (Figure 7).

The interaction between mycobacteria and host cell inflammasomes involves a delicate balance of multiple sensors and bacterial effectors [22]. ESX-1–mediated cytosolic access has emerged as a central requirement for inflammasome activation during mycobacterial infection [23,24]. While EsxA has been extensively studied in this context [16,25–28], retained IL-1β production in ESAT-6–deficient or secretion-impaired strains like H37Ra has suggested the existence of additional ESX-1 effectors that contribute to inflammasome signaling [17]. Our systematic screening identifies EspF as a dominant NLRP3 activator, providing a mechanistic explanation for this long-standing observation and extending the current model of ESX-1–dependent immune modulation beyond ESAT-6 alone. Moreover, the strong conservation of EspF among pathogenic mycobacteria, including *M. tuberculosis*, *M. bovis*, and *M. marinum*, suggests that inflammasome manipulation by EspF represents a conserved virulence strategy rather than a species-specific phenomenon.

Mechanistically, our data demonstrates that EspF directly binds to the NACHT and LRR domains of NLRP3, which govern ATP-dependent oligomerization and autoinhibitory regulation, respectively [28,29]. This binding profile mirrors that of mycobacterial PPE13, which targets identical domains to facilitate inflammasome assembly reported in our previous study [30]. By binding with these domains, EspF likely stabilizes NLRP3 in its “open” active conformation or facilitates its transition from an autoinhibited state. Such direct targeting of the core sensor is a sophisticated strategy shared by diverse pathogens, including *Shigella* Type III Secretion Effector IpaH4.5 and influenza PB1-F2 [31,32]. Interestingly, recent research has also identified host factors like NLRP11 as being essential for the efficient activation of canonical NLRP3 during mycobacterial infection in human macrophages, further complicating the host-pathogen crosstalk [33]. These findings suggest that manipulating the inflammasome is a highly conserved strategy for immune evasion or remodeling.

NLRP3 inflammasome activation is tightly controlled by post-translational modifications (PTMs), particularly ubiquitination [34]. Basal ubiquitination maintains NLRP3 in an inactive state, whereas deubiquitination or specific non-degradative ubiquitin linkages promote inflammasome assembly. Accordingly, multiple E3 ubiquitin ligases have been implicated in NLRP3 regulation, functioning either as negative regulators through K48-linked ubiquitination and proteasomal degradation or as positive regulators that facilitate inflammasome activation via K63-linked or non-canonical ubiquitin chains [35]. TRIM25, a member of the tripartite motif (TRIM) family of E3 ubiquitin ligases, is best known for its role in antiviral immunity, particularly in RIG-I–mediated type I interferon responses [20]. Since its discovery as a pivotal regulator of the cytosolic RNA sensor RIG-I, numerous studies have established TRIM25 as a central hub for coordinating innate defenses against various viral pathogens [36]. Specifically, TRIM25 facilitates the K63-linked ubiquitination of RIG-I CARD domains, a step that is essential for signal transduction to the mitochondrial adaptor MAVS. The recent evidence identifying TRIM25 as a significant positive regulator of porcineNLRP3 inflammasome by enhancing the pro-caspase-1 interaction with ASC, a function notably targeted for subversion by the NS1 protein of the 2009 pandemic influenza A virus [37]. Here, we identify a previously unappreciated role for TRIM25 in inflammasome regulation during bacterial infection. We demonstrated that TRIM25 interacts with EspF and is indispensable for EspF-driven IL-1β maturation, caspase-1 activation, and pyroptosis. Moreover, TRIM25 directly interacts with NLRP3 to facilitate the K63-linked ubiquitination of NLRP3, and this interaction is enhanced by EspF in a dose-dependent manner. These findings support a model in which EspF functions as a molecular scaffold that promotes recruitment of TRIM25 to the NLRP3 complex, thereby facilitating inflammasome activation. Beyond TRIM25, multiple TRIM family members have been shown to regulate NLRP3 in opposing directions. For instance, TRIM31 serves as a critical E3 ligase whose promotion of NLRP3 activation is intricately modulated by the immunoproteasome, illustrating a sophisticated layer of proteasomal control over innate signaling [38,39]. In addition, TRIM50 has been identified as a direct facilitator of NLRP3 oligomerization, effectively bypassing certain upstream priming requirements to accelerate inflammasome assembly [40]. The pathological significance of these regulators extends to sterile inflammation, where TRIM62 is reported to orchestrate NLRP3-regulated neuroinflammation, contributing to secondary tissue damage in ischemic injury models [41]. Collectively, these studies, along with our discovery of the EspF-TRIM25 axis, underscore the diverse strategies through which the TRIM family fine-tunes or hijacks inflammasome-dependent immunity.

Finally, our data indicate that EspF-mediated inflammasome amplification benefits the pathogen in our models. Although IL-1 signaling is required for resistance to TB, excessive inflammasome activation and lytic death can promote tissue injury and bacterial spread [22]. EspF enhanced GSDMD cleavage and pyroptosis in macrophages and increased lung pathology and bacterial burden *in vivo* with recombinant BCG, consistent with an inflammation-driven permissive niche rather than improved clearance. This stands in instructive contrast to reports such as EST12, which has been linked to pyroptosis-associated immune pathways that increase clearance in some settings, highlighting that the net outcome of inflammasome engagement likely depends on timing, bacterial load, host cell types, and the balance between bactericidal containment vs destructive immunopathology [42]. Our *in vivo* analyses relied on recombinant BCG rather than virulent Mtb, and future studies using EspF-deficient or separation-of-function mutants in Mtb will be required to define the contribution of EspF across different stages of infection, including latency and reactivation.

In summary, we identify EspF as a novel and conserved ESX-1 effector that directly targets the NLRP3 inflammasome through a TRIM25-dependent mechanism. Together with prior work describing additional mycobacterial inflammasome-modulating effectors, our findings support a model in which pathogenic mycobacteria deploy mechanistically diverse strategies to fine-tune inflammasome signaling toward outcomes that can favor persistence and pathology. These insights advance our understanding of mycobacterial virulence mechanisms and highlight inflammasome regulatory nodes, including the EspF–TRIM25–NLRP3 axis, as potential targets for host-directed therapeutic intervention in tuberculosis.

## Supplementary Material

Supplemental Material

Supplemental Material

Supplemental Material

## Data Availability

All data that support the findings of this study are present in the paper and the Supplementary figure. Raw data associated with this article have been deposited in the Figshare repository (https://doi.org/10.6084/m9.figshare.31143826).
